# Perceived guideline clarity impacts guideline-concordant care for breast cancer screening in women age 40–49

**DOI:** 10.1186/s12905-023-02190-w

**Published:** 2023-02-20

**Authors:** Michelle B. Nadler, Ann Marie Corrado, Brooke E. Wilson, Alexandra Desnoyers, Eitan Amir, Noah Ivers, Laura Desveaux

**Affiliations:** 1 Division of Medical Oncology and Hematology, Department of Medicine, Princess Margaret Cancer Centre, 700 University Avenue, Toronto, ON M5G 1Z5 Canada; 2 Department of Medicine, University of Toronto, Toronto, ON Canada; 3 The Peter Gilgan Centre for Women’s Cancers, Women’s College Hospital, Toronto, Canada; 4 Kingston Health Sciences Centre, Kingston, ON Canada; 5 University of Lavel, Quebec City, QC Canada; 6 Women’s College Hospital, Toronto, Canada; 7 Department of Family and Community Medicine, University of Toronto, Toronto, Canada; 8 Trillium Health Partners, Toronto, Canada

**Keywords:** Breast cancer screening, Mammogram, Age 40–49, Guidelines, Barriers and facilitators, Guideline implementation, Theoretical domains framework

## Abstract

**Background:**

Canadian and US Task Forces recommend against routine mammography screening for women age 40–49 at average breast cancer risk as harms outweigh benefits. Both suggest individualized decisions based on the relative value women place on potential screening benefits and harms. Population-based data reveal variation in primary care professionals (PCPs) mammography rates in this age group after adjusting for sociodemographic factors, highlighting the need to explore PCP screening perspectives and how this informs clinical behaviours. Results from this study will inform interventions that can improve guideline concordant breast screening for this age group.

**Methods:**

Qualitative semi-structured interviews were performed with PCPs in Ontario, Canada. Interviews were structured using the theoretical domains framework (TDF) to explore determinants of breast cancer screening best-practice behaviours: (1) risk assessment; (2) discussion regarding benefits and harms; and (3) referral for screening.

**Analysis:**

Interviews were transcribed and analyzed iteratively until saturation. Transcripts were coded deductively by behaviour and TDF domain. Data that did not fit within a TDF code were coded inductively. The research team met repeatedly to identify potential themes that influenced or were important consequences of the screening behaviours. The themes were tested against further data, disconfirming cases, and different PCP demographics.

**Results:**

Eighteen physicians were interviewed. The theme of perceived guideline clarity (a lack of clarity on guideline-concordant practices) influenced all behaviours and moderated the extent to which the risk assessment and discussion occurred. Many were unaware of how risk-assessment factored into the guidelines and/or did not perceive that a shared-care discussion was guideline-concordant. Deferral to patient preference (screening referral without a complete discussion of benefits and harms) occurred when the PCPs had low knowledge regarding harms and/or if they experienced regret (TDF domain: emotion) resulting from prior clinical experiences. Older providers described patient’s influence impacting their decisions and physicians trained outside Canada, practicing in higher-resourced areas, and female physicians described being influenced by beliefs about consequences of benefits of screening.

**Conclusion:**

Perceived guideline clarity is an important driver of physician behaviour. Improving guideline concordant care should start by clarifying the guideline itself. Thereafter, targeted strategies include building skills in identifying and overcoming emotional factors and communication skills important for evidence-based screening discussions.

**Supplementary Information:**

The online version contains supplementary material available at 10.1186/s12905-023-02190-w.

## Introduction

The Canadian Task Force for Preventive Health Care and the United States Preventive Services Task Force recommend screening mammography every 2–3 years for women age 50–74 at average breast cancer risk based on evidence of reduced breast cancer-specific mortality [1–3]. Both recommend against routine screening for women age 40–49 due to concerns that the harms (psychological, false-positives, and overdiagnosis) outweigh the benefits; however, both guidelines state that the decision to start screening in this age group should be individualized, ‘conditional on the relative value a woman places on possible benefits and harms from screening’ [1, 3]. Radiology societies have published recommendations or advocated that screening should start routinely at age 40 or 45 [4–6], citing updated research showing a greater mortality benefit and less frequent over-diagnosis, suggesting that benefits outweigh harms [6, 7]. There is significant controversy in the literature over the conflicting nature of these two published recommendations.

For Ontario women aged 40–49, a screening mammogram is covered by the Ontario Health Insurance Plan (OHIP) if ordered by a physician or nurse practitioner [8], therefore a referral from a primary care provider (PCP) is required to access publicly funded screening mammography in this age group. Prior subjective and objective reports of variation in practice suggests that guideline concordant care is not implemented consistently in this age-group or others, ie that there is variation among PCP breast screening practices [9–12].

Previously, we explored and reported on the determinants (barriers/facilitators) of variation amongst family physicians related to five behaviours we defined as necessary to support guideline-concordant care for women aged 40–49: (i) breast cancer risk assessment; (ii) discussion of benefits, harms, and preferences (ie ‘shared decision-making’ SDM); (iii) decision/referral for screening mammogram; (iv) genetics referral; and (v) high-risk screening enrolment [13]. Physicians reported low knowledge regarding breast cancer risk factors and criteria for genetic testing, lack of skills to synthesize risk, and described that a formal risk assessment is rarely a part of their clinical management [13]. A lack of support, time, absence of reminder services, uncertainty about the efficacy of screening, and perceived conflicting evidence about screening were also influencing factors [10, 14].

In the current study, the primary goal was to identify common themes, that could help explain contextual factors (causes) or consequences of variation in screening mammography behaviours. We focused on the first three screening behaviours as they are most clearly part of the guidelines. As a secondary aim, we explored how these themes varied across physician demographics. This work can help inform matching of physicians to potentially effective implementation strategies that might increase guideline concordant breast screening for those aged 40–49.

## Methods

### Study design, data measures, & data collection

Overall study design and data collection methods have been previously described and COREQ checklist followed (Supplementary material) [13]. Briefly, one-on-one semi-structured telephone interviews were conducted with a sample of primary care physicians in the Greater Toronto Area, Canada between January and November 2020. Interviews were de-identified and professionally transcribed. The interview guide and initial analysis of barriers and facilitators was structured and informed by the theoretical domains framework (TDF). The TDF is a theory-informed, comprehensive determinant framework used to examine the underlying determinants (i.e., barriers/facilitators) for specific behaviour(s) [15, 16]. Ethics approval was obtained at Women’s College Hospital # 2019-0141-E.

During the interviews, it became apparent that asking about “the guidelines” was confusing to participants; therefore, the interview guide was modified to ask about routine practice relating to mammography in this age group, and this was followed up with TDF-based questions about their practice and the behaviours of interest. Once this part of the discussion was completed, the specific phrasing of the Canadian Task Force key recommendation for breast screening guidelines for age 40–49 were read to participants and their reactions and opinions to this were captured. The interviewer (MBN) introduced herself as a breast cancer medical oncologist. Her reflexivity was to remain as neutral as possible with the view of understanding each participant’s clinical practice, experience, and reasons for this. She would normalize all participant’s questions or concerns in a neutral tone, ie stating that in her clinical experience, she had seen several different approaches to screening, that there was variation amongst participant answers, no ‘wrong’ answers. MBN stated that the goal of the study was to try to understand reasons behind each physician’s clinical practice. The second coding author (AMC) is a project manager and qualitative researcher, with expertise in women’s health. The project team has expertise in screening data and guideline implementation. When both coding authors did not find any new information related to the research question in 2–3 consecutive transcripts, the team sought disconfirming cases by interviewing physicians in under-represented demographic categories (men and lower-resourced areas). Following 3 additional interviews of these participants, the team concluded saturation was reached as the themes continued to explain accurately the screening behaviours and drivers of variation.

### Data analysis

First, we established an operational definition of guideline-concordant mammography screening in this age group as including (i) a breast cancer specific risk assessment and (ii) an informed discussion of benefits and harms of screening mammography. If the physician determines that screening is appropriate (benefits likely greater than harms) but the patient chooses not to screen, this was considered guideline-concordant, as patients have the right to decline investigations. If the physician believes that screening is inappropriate (harms likely greater than benefits), the physician should recommend against screening; however, if such a patient expresses a desire for screening, a referral may still be considered guideline-concordant after eliciting patient values, discussion of benefits and harms, and obtaining informed consent.

A pragmatic, directed content analysis approach was taken whereby the data was deductively coded to corresponding TDF domains representing barriers and facilitators of the screening behaviours [17, 18]. Interview transcripts were coded independently by two members of the research team (MBN & AMC), coded first by behaviour of interest and then by the identification and application of the relevant TDF code. If the researchers felt a piece of transcript was relevant to the research question or modified a screening behaviour, but did not fit within a TDF domain, it was coded inductively. In the initial analysis, individual behaviours were assess for the presence of specific TDF-barriers and facilitators to understand influences on each screening behaviour. For this analysis, the research team sought to identify common themes that acted as contextual factors (causes) or consequences of variation in the screening behaviours. The research team met repeatedly to review all inductive codes, which included definitions and a list exemplar quotations. The list of inductive codes were discussed, compared to potential discomfirming cases or variation, and narrowed until they either applied to more than one screening behaviour as either an overarching driver of behaviour or helped explain the influence or consequence of some of the TDF domains.

For our secondary aim, a framework approach was used to explore whether demographic characteristics explained sources of variation within the dataset for the third behaviour of decision and referral for mammography [19]. A priori demographic variables and TDF-domains for analysis were informed by the literature and prior work. Demographic variables included provider sex, location of practice (urban versus sub-urban), Canadian versus non-Canadian medical graduate, and age (above or below average). For location of practice, providers from Toronto, Thornhill, and North York were grouped together as high-resourced, as these providers described their practices as being located in or directly adjacent to the high resourced environment within Toronto, Ontario. Providers from Scarborough, Brampton, Pickering, Ajax, and Orangeville were categorized as lower resourced environments as these areas are further outside Toronto and providers described access to resources within Toronto but a lack of similar resources locally (and/or their patients had increased barriers to accessing the Toronto-based resources). The TDF domains chosen for the framework analysis included social influence—radiologists, social influence- patient, emotion, beliefs about capabilities, and beliefs about consequences because they were the most common domains to influence the third screening behaviour across a subset of participants. Our initial analysis found that the TDF-code “social influence” could be applied coming from both patients and radiologists, so these were coded as distinct barriers for the coding and analyses. The presence or absence of the determinants of interest were categorized across the transcripts. Only determinants that had the same direction of association were counted (for example, social influence of radiologist had to be that this influence favoured the screening behaviour). When the expression of a determinant was more heavily present for one demographic group compared to another (e.g., males versus females), the difference was reported.

## Results

### Participants

Twenty providers expressed interest in participation. Of these, two were not interviewed as their demographic category was already saturated. Mean age was 48 years and 72% identified as women (see Additional file 1: Table S1). Interviews lasted approximately 30–45 min.

### Themes

There were two clear themes that influenced multiple behaviours and moderated the influence of TDF determinants. "Perceived guideline clarity,” defined as the physician being uncertain what the guidelines recommended, was a contextual or causal factor that caused PCP not to follow guideline-concordant behaviours. “Deferral to patient preferences,” defined as a decision about screening without an informed discussion, was an over-arcing consequence of the TDF-based screening barriers. PCP who had low knowledge or emotion often did not present women with comprehensive information on screening benefits and harms. Supporting quotations can be found in Table 1.

**Table 1:** Supporting quotations for themes of perceived guideline clarity and deferral to patient preference

Behaviour & description	Supporting quotes for contextual factor of perceived guideline clarity	Supporting quotations for contextual factor of deferral to patient preference
General statements about perceived guideline clarity Physicians describe general confusion about what the guidelines recommend (first quote), confusion about which women the guideline applies to (second quote), and confusion between criteria for genetic testing versus screening mammography (third quote)	*“Mm-hmm, yeah very, very vague, you know, you just read that, over and over and … It’s not clear… my feeling after reading the guideline a few times was like just do whatever you have to do. – P13* “M*y understanding of guidelines is that unless they’re in a high-risk group, it’s not recommended between ages 40 and 49… you need about three relatively first or second-degree relatives to be able to do that.” – P18* *“When I send patients {to genetics} who I think are high risk and could potentially warrant earlier screening, and they’re not, I’m often surprised… I think I’m doing the right thing with my counselling. Because even for the patients who I think should have it, when genetics says no, they are not high risk than the average population despite you know, your mother at a young age having breast cancer they’re reassured I guess after speaking to the expert” -P14*	
Behaviour 1: risk assessment Physicians describe that they do not perceive that the guidelines suggest to do a breast cancer risk assessment (first quote) and that if they do a risk assessment, the calculated risk is not tied to a specific management strategy (second quote)	*“I think intuitively, you should be assessing risk with any decision that you make, obviously. But not – no, the guidelines, to me, did not point me towards something like the IBIS or anything like that… in terms of the actual numbers and what not, again, I had to kind of learn them myself.” -P20* *“Yes, otherwise I think [the risk assessment] is useless… it spews something out to me but I don’t know how to interpret it or what the next step are… But if there was something… similar to the Framingham or Osteoporosis guidelines… if there’s kind of an interpretation of what to do once it spews out the [risk] value… [where] I know what to do when it’s low, moderate, or high risk.”* -P14	
Behaviour 2: shared-care discussion Physicians may engage in shared-care discussion (or not), but either way do not feel the guideline does not inform whether shared-care should be done Physicians describe two situations where they defer to patient preference without complete informed discussion of potential screening harms	*“It [the guideline] doesn’t tell you anything… it doesn’t tell you what the risks are, the benefits or how to counsel them… It puts a lot of emphasis on the patient value which I don’t think it’s fair. I think most patients if they’re in to see the doctor are valuing their health in some sort of way. And not helpful to the doctor or to the system” –* P14 *“I think it’s probably good just to start these conversations early, but that statement doesn’t help me very specifically. It doesn’t tell me that I should have those conversations early. I think that’s just my personal feeling that I should do it that way.”* – P08	*“So if I thought her risk was high, then I would really push. And if I thought her risk was very low then I would spend a couple of minutes saying, ‘Listen, I really think you’re okay. There is a tiny bit of radiation and radiation is cumulative.’ But at the end of the day, I listen to peo’le and if somebody really wants a mammogram, you never know why they really want it, so I let them have it.” – P05* *“If somebody brings it up and they are a bit adamant about getting it done. They’ve had a friend that has it. They’re anxious about it. They hear that the Americans do it. I don’t hold back, and I will be happy to do it.” -P01*
Behaviour 3: decision/referral for screening mammography Physicians state that guidelines do not explain which benefits and harms should be discussed. They describe following radiology guidelines rather than the Task Force guidelines Physicians describe situations where they recommend screening due to past experiences (TDF domain: emotion)	*“Like, what does that mean? …many patients will say, “What should I do?”.. and most practitioners don’t understand what the relative risks and benefits and can’t even have that conversation.”* -P04 “*The medical post had a blurb from Dr. XX, the Head of Mammography… she said that the Canadian Task Force was flawed, that the people on the panel weren’t mammographers… And I believed her, she had good data, she does this every day. She said the best screening was every year for 40–50. And yes, there will be false positives but you're going to save lives… So I think that the hard evidence probably supports doing it. The economic evidence doesn't. And I'm sticking with the head of the [City] Hospital versus the Canadian Task Force”* -P05 *“So, it’s frustrating, I don’t actually know what the right thing is to do, but I’m starting to add in ultrasound anyhow” – P17*	“*So, I think you screen. I know it’s certainly not guideline-based, but I find it really hard to extrapolate guidelines to a person sitting in front of me. And you know, we all know women in their forties that have been diagnosed with breast cancer, they all have stories, and those stories are pretty impactful.” -P03* *“It’s very hard to tell someone they can't have something and then take on the burden of, oh, I hope they don’t develop breast cancer at forty five and I'm the one that told them not to do it… it’s such an infrequent request in my population, that I usually will do the education, discuss the problems. Most of the time they're not interested in it, then if they are still are, I will order a mammogram.”* – P02

“Perceived guideline clarity” was defined as the physician being uncertain what the guidelines recommended. This included statements made pertaining to uncertainty about who the guidelines applied to, what the conditional statement meant, what physician behaviours were considered guideline concordant, and how providers should counsel patients. This was a dominant theme that affected almost every physician interviewed: a group of physicians who appeared to practice the three guideline-concordant behaviours listed stated they learned these behaviours elsewhere (or felt this was the best way to interpret the guideline) but still commented that the guideline was confusing and the other group of physicians discussed overall confusion and lack of guideline clarity related to some or all screening-behaviours.

“Perceived guideline clarity” affected breast cancer risk assessment, as many physicians were not aware that assessing lifetime breast cancer risk would be a necessary step to determine if the guidelines applied to the patient. This caused some PCP to apply a guideline for women at average-risk of breast cancer to women who may have high lifetime risk of breast cancer, who should be enrolled in a high-risk screening program. Physicians who performed a risk assessment (formally or informally) often confused the criteria for genetic testing with guidelines for screening, so if a woman was told she didn’t qualify for genetic testing, the physician believed she also “did not qualify” for a screening mammogram and would not present this as an option. This theme impacted PCP knowledge of risk factors, risk-assessment tools, and awareness of different levels or categories of risk. Providers who perceived that the guidelines did not provide clarity regarding different management options for levels of risk were more likely to hold the belief about consequence that performing a risk assessment wouldn’t change their management.

“Perceived guideline clarity” influenced the behaviours of both discussion and referral. Some physicians did not interpret that the guidelines suggested that a shared-care discussion should occur with all women in this age group or that all women in this age group should have a choice about screening. Others felt that the word ‘value’ was impractical, as they considered that all women in their practice would value their personal health and accept any screening offered to them. Physicians seemed to feel uncomfortable recommending against screening or not referring for mammography when they felt the guidelines were unclear or they placed more emphasis on the individual patient-level value of screening within the guidelines. Some providers questioned the credibility of the guidelines, having more faith in the recommendations of radiologists (TDF domain: social influence—radiologist). This lack of clarify and question about credibility led some physicians to recommend management options that are clearly not evidence based (due to more harm than benefit at the population level), such as screening breast examination or breast ultrasound.

“Deferral to patient preference” was defined as a decision about screening without an informed discussion. Physicians often described acquiescing to any screening request due to an outside influence or past experience rather than providing objective information (benefits and harms) for a woman to make an informed choice based on her values. Some of these women may then experience over-diagnosis (and over-treatment) without a priori being made aware of this possible harm.

Physician narratives suggest that they tried to provide as much information as possible to inform patients; however, some were limited by the knowledge of what benefits and harms to discuss, and others by the skills to explain to patients when or why screening is not routinely recommended. Without this knowledge or skill, physicians seemed more likely to yield to the patient’s preference to screen without a fully informed discussion. This decision was often moderated by TDF code of emotion: physicians described potential anticipated regret for a case where they recommended against screening mammogram if that woman later developed a breast cancer. Similarly, physicians with a prior experience of a patient in this age group with a clinically-detected (rather than screen-detected) cancer sometimes drew the (inappropriate) conclusion that the outcome would have certainly been improved through screening.

### The interaction of demographic characteristics and behavioural drivers

Physicians with certain demographic features seemed to be differentially influenced by specific TDF domains (See Table 2). Older physicians were more likely to describe TDF domains of emotion and social influence of the patient as influences on their decision to order screening mammography. They tended to recognize how past clinical cases influenced their current behaviour, often describing this as relying on their ‘clinical experience.’ In this way a past experience acted as an availability heuristic or bias which appeared to increase the likelihood of making a non-guideline-concordant screening decision. Physicians who identified as female were also more likely to describe emotion as influencing their decision to screen, although there was no clear reason described for this within their narratives.

**Table 2:** Interaction of demographic characteristics and behavioural drivers

Demographic characteristic	TDF domain and description	Supporting quotations
Age	Older physicians were influenced to screen based on TDF domains of *Emotion* (past negative experience) and *Social influence* of the patient Younger Physicians described the TDF domain facilitator of *Beliefs in Capabilities* to explain when screening not recommended	*“You know, over years, I’ve been in practice for 16 years… based on the positive cases that I saw in real life, I don’t believe that we have to wait until age 50 to start the screening. I saw lots of women that, you know, we missed or we diagnosed Grade 3 definitely below age 50. So, my belief in, based on my clinical experience is that age 50 for start screening is too late.” -013* *“I would say 98% of the women I’ve spoken to, as long as I sit down and give them a proper explanation* [of why screening not recommended routinely]*, and sometimes I would even refer them to Task Force. Most of them were very satisfied and don’t bring it up again.” – P16*
Gender	Physicians who identified as female described TDF domain barriers of *Emotion & Beliefs about Consequences*	*“It’s been proven to be so with my clinical experience all this year. And just like when they’re younger, we miss them. I just find it quite devastating. They just literally have more [tortuous] difficult journey than people older. So I’m just totally sold on that.” -P09*
Location of medical training	Physicians with Canadian Training described *Beliefs about Capabilities* to discuss why screening not routinely recommended Non-Canadian Trained physicians held the *Beliefs about Consequences* that not screening would lead to inappropriate outcomes	*“And that’s kind of been drilled into our heads…choosing wisely… our generation is probably a little bit more mindful… it’s minimal testing for things that are not guideline” -P14* *“I’m conscious that everything should be evidence-based on guidelines but I just feel like it’s a little too late. I always do let people know that at least previously the guidelines in the States sort of were to start at age 40… there’s some degree of financial conservatism driving those guidelines in Canada, potentially.”- P02*
Geographic area	Physicians working in higher resourced areas descrived *Beliefs about consequences* that patients still want screening even if harms were discussed; physicians working in lower resourced areas described the *beliefs in capabilities* to explain why screening not recommended	*“I work in a pretty well-educated and affluent area, and I find people are pretty pro early screening..”. – P06* *“I think with my training… not wasting resources doing things; I had some really good attendings that would question ‘are you doing something because that’s what you were taught or are you doing it because there’s actually research behind it’ and that was one of the things that really carried through with my practice” -P16*

Physicians who received their medical education outside Canada, as well as physicians practicing in higher-resourced areas, and female physicians appeared more strongly affected by TDF domain of beliefs about consequences of screening compared to their counterparts. These groups tended to overestimate benefits and/or underestimate the harms of screening; this led to a recommendation to screen despite the fact that the benefit to harm ratio appeared to be unfavourable.

Physicians who were younger, Canadian medical graduates, or practicing in lower resourced settings all had higher beliefs in their capabilities to explain why screening was not recommended. They sometimes described the “Choosing Wisely” campaign [20] as the rationale to guide this decision. Some providers in sub-urban locations described the under-resourced nature of their location relative to urban locations as driving their ‘choosing wisely’ decisions in order to appropriately allocate resources.

## Discussion

Two key themes, perceived lack of guideline clarity and deferral to patient preference, play central roles in breast screening behaviours in primary care for women aged 40–49. The former is a contextual factor that moderates screening behaviours and the latter is an over-arching consequence of the TDF-screening barriers.

Perceived guideline clarity impacted screening-related behaviours and many of the determinants of these behaviours. This acted as an overarching barrier to guideline-concordant care, especially for the physicians who felt the guidelines were not credible and instead trusted the advice of radiologists. Within the literature, primary care clinicians report that lack of consensus within the medical community and discordant or divergent guidelines are a significant barrier to appropriate care [10, 21]. In situations of guideline uncertainty for cancer screening, family physicians are more likely to order screening tests when patients have anxiety about cancer, expectations about receiving tests, or when they believe there is more benefit than harm [22]; many of these situations, thoughts, and beliefs were described by participants in our study. The guidelines are fairly clear to suggest no routine screening as a primary recommendation, but do not provide any guidance regaring in whom screening may be considered as the exception(s).The Canadian Task Force guideline does not indicate anything about an individualized breast cancer risk assessment or routine discussion with a suggested frequency of when to perform it, nor provide recommendations regarding how to act based on different numerical values from such assessments, yet this would seem to be a necessary step in order to reflect on potential benefits and harms of screening. Our findings suggest that if guideline specifically stated that certain behaviours (such as performing a risk assessment and having a shared-decision making discussion) were recommended, this could help decrease some providers’ confusion regarding optimal care.

The Canadian Task Force defines shared-decision making as a structured process to incorporate evidence as well as patient values and preferences into screening decisions [23]. Importantly, the task force specifies that SDM is not about giving the patient whatever test or treatment they request or about leaving the patient to decide on their own. Although some PCP provided appropriate SDM, the theme of deferral to patient preference describes situations when women chose a course of action (typically screening) without being fully informed or when physicians over-estimated the benefit or under-estimated the harms of screening during the SDM discussion. The latter can occur despite a physician’s best intention to perform SDM appropriately and is a common finding in the SDM literature [24]. Within behavioural science this is variably described as a “therapeutic illusion” (“an unjustified enthusiasm for treatment on the part of both doctors and patients” [25] or “commission bias” (the tendency toward action rather than inaction) [26]. This resonates with narratives that some physicians provided in our study about the certainty of benefit (ie beliefs about consequences) with screening. Additionally, the concepts of “loss aversion bias” or “anticipated regret” which describe the fear of missing something (and belief that the outcome would have been different if it was caught earlier) were observed as ways in which the TDF domain *emotion* may interact with the domain beliefs about consequences and lead to guideline discordant care due to over-estimation of potential benefits. It is possible that increased exposure or increased relatability regarding these undesirable events may increase the likelihood of anticipated regret, therapeutic illusion, or commission bias; this may explain some of the variation observed across physicians. In particular, female physicians in our study tended to over-estimate benefits of screening. Within the general breast screening literature (age 50–74), female physicians order screening mammography and pap smears more frequently [27, 28] whereas male physicians order more prostate-specific antigen screening tests [29] and have higher beliefs in their effectiveness for screening [30]. Taken together, our findings suggest that a clear list of benefits and harms, approximate incidence, and language for discussion as part of SDM could help physicians to more accurately estimate each woman’s individual benefit:harm ratio for screening. This would ensure the physicians provided complete informed consent and help them to to guide women toward a final screening decision (a necessary component of SDM, [23]). Increasing knowledge and awareness of the biases and associations described in our study may help physicians assess the benefit:harm ratio more objectively.

As noted above, our findings provide the basis for a few recommendations. Given both the lack of guideline clarity (and/or suboptimal guideline credibility for some), a statement outlining which specific behaviours are required to be guideline concordant is important. This could include statements that indicate that shared-care discussions are required with all women. Given that many PCP are aware of the conflicting advice between the Task Force and Canadian Association of Radiologists, and do not know which to follow, a consensus statement including minimum behaviours or concepts for which there is broad support, could help decrease the variation in care at the provider level. Our results suggest that a checklist of pertinent benefits and harms of screening with definitions would improve knowledge and comprehensive shared-care discussions. Targeted strategies include building skills in identifying and overcoming emotional factors and communication skills important for evidence-based screening discussions. The theme “deferral to patient preference” highlights awareness of the harms that can occur when guidelines are not followed: women receiving tests they may not want or receiving a test that leads to a harm they were not informed about. While it is important to highlight that guidelines suggest all women should have the choice to screen, it is still inappropriate to do so without discussion and informed consent about potential harms.

### Limitations

This study has limitations, many of which are described in our prior work [13], including that guideline-concordance could not be confirmed, the axiology of the researcher (a pragmatist to try to understand the issues of guideline-concordance), and our recruitment was limited to one major urban centre. The issue of comparing higher- versus lower- resourced settings warrants further exploration and explanation. Despite trying to recruit broadly, we may have interviewed physicians with more divergent views and practices; however, the contextual factors applied to many physicians across the practice spectrum. Conducting the interviews via telephone may have limitd the ability to interpret contextual and nonverbal data, which may have suggested more nuanced responses. Finally, the associations with demographic factors within this qualitative study should all be viewed as hypothesis-generating.

## Conclusion

Contextual factors of perceived guideline clarity and deferral to patient preference impacted several behaviours and TDF-moderators of behaviour in our study. These factors need to be considered when informing system-wide initiatives to support primary care physicians in guideline-corncordant breast cancer screening in those aged 40–49.

## Supplementary Information

**Additional file 1**: **Table S1**. Demographic information of participants

## Data Availability

De-identified datasets used and analysed during the current study are available from the corresponding author on reasonable request. Every effort to maintain and safeguard the privacy and confidentiality of participants will be maintained.

## Abbreviations

OHIP: Ontario health insurance plan
PCP: Primary care provider
SDM: Shared-decision making
TDF: Theoretical domains framework
