# Supplementary material for: Perceived guideline clarity impacts guideline-concordant care for breast cancer screening in women age 40–49
Source: BMC Womens Health. 2023 Feb 20;23:75. doi: 10.1186/s12905-023-02190-w (PMC9942408; doi:10.1186/s12905-023-02190-w)
Supplement: Supplementary file 1 — Additional file 1: Table S1. Demographic information of participants [file 12905_2023_2190_MOESM1_ESM.docx]

Supplementary Table 1: Demographic Information of Participants

| **Demographic** | **Participants n (%)** |
| --- | --- |
| Female  Male | 13 (72%)  5 (28%) |
| Age; average (range) | 48 years (33-65) |
| Location | Toronto: 8 (44%)  Thornhill: 2 (11%)  North York: 2 (11%)  Sub-Urban*: 6 (33%) |
| Number of Physicians in Practice | - 1. Physicians: 10 (56%)   6-10 Physicians: 6 (33%)  10-20 Physicians: 1 (5%)  > 20 Physicians: 1 (5%) |
| Estimated Practice Size (patients):  Average (range) | 1690 (800 – 3000) |
| Estimated Patients Seen Weekly  Average (range) | 123 (60 – 250) |

*Sub-Urban includes Orangeville, Vaughan, Scarborough, Brampton, Pickering, and Ajax
